# Identification and validation of a metabolism-related gene signature for predicting the prognosis of paediatric medulloblastoma

**DOI:** 10.1038/s41598-024-57549-2

**Published:** 2024-03-30

**Authors:** Jun Su, Qin Xie, Longlong Xie

**Affiliations:** 1 Department of Neurosurgery, The Affiliated Children’s Hospital Of Xiangya School of Medicine, Central South University (Hunan children’s hospital), No. 86 Ziyuan Road, Changsha, 410007 Hunan China; 2 Department of Neurosurgery, Xiangya Hospital, Central South University, No. 86 Xiangya Road, Changsha, 410008 Hunan China; 3 Pediatrics Research Institute of Hunan Province, Hunan Provincial Key Laboratory of Pediatric Orthopedics, The Affiliated Children’s Hospital Of Xiangya School of Medicine, Central South University (Hunan children’s hospital), No. 86 Ziyuan Road, Changsha, 410007 Hunan China

**Keywords:** Medulloblastoma, Metabolic-related genes, Ornithine decarboxylase, Migration and invasion, Cell biology, Cancer metabolism

## Abstract

Medulloblastoma (MB) is a malignant brain tumour that is highly common in children and has a tendency to spread to the brain and spinal cord. MB is thought to be a metabolically driven brain tumour. Understanding tumour cell metabolic patterns and characteristics can provide a promising foundation for understanding MB pathogenesis and developing treatments. Here, by analysing RNA-seq data of MB samples from the Gene Expression Omnibus (GEO) database, 12 differentially expressed metabolic-related genes (DE-MRGs) were chosen for the construction of a predictive risk score model for MB. This model demonstrated outstanding accuracy in predicting the outcomes of MB patients and served as a standalone predictor. An evaluation of functional enrichment revealed that the risk score showed enrichment in pathways related to cancer promotion and the immune response. In addition, a high risk score was an independent poor prognostic factor for MB in patients with different ages, sexes, metastasis stages and subgroups (SHH and Group 4). Consistently, the metabolic enzyme ornithine decarboxylase (ODC1) was upregulated in MB patients with poor survival time. Inhibition of ODC1 in primary and metastatic MB cell lines decreased cell proliferation, migration and invasion but increased immune infiltration. This study could aid in identifying metabolic targets for MB as well as optimizing risk stratification systems and individual treatment plans for MB patients via the use of a metabolism-related gene prognostic risk score signature.

## Introduction

Medulloblastoma (MB), which accounts for 10 to 25% of paediatric tumours, is the most common malignant brain tumour in children, and it has a tendency to spread to the brain and spinal cord^1^. The strategies for treating MB include surgical resection, chemotherapy, and radiotherapy, and these strategies are tailored based on clinical radiological risk criteria^2^. Currently, the 5-year survival rate of patients with MB is 70–80%, which is better than the survival rate in previous years^3^. Although some MB patients achieve favourable therapeutic outcomes, they often suffer from neurological and endocrine sequelae^4^. Recently, with advances in molecular genomics, some breakthroughs have been made in understanding the genetic background of MB^5^. According to DNA methylation data, gene expression profiles, or proteomics data, MB can be classified into four molecular subtypes: WNT, SHH, Group 3, and Group 4. The molecular subtypes are associated with the prognosis of MB; however, patients within the same subgroups exhibit considerable heterogeneity, and the Group 3 and Group 4 subtypes of MB remain difficult to distinguish in clinical practice. Therefore, identifying sensitive and specific markers and constructing new predictive models are urgently needed to improve the prognosis of patients with MB.

Metabolic features are known to determine the phenotypes of tumour cells. The activation of oncogenes and deletion of tumour suppressors contribute to metabolic reprogramming in tumours. Currently, common metabolic patterns in tumours include abnormalities in lipid synthesis, aerobic glycolysis, oxidative phosphorylation, and amino acid metabolism. Accumulating evidence indicates that the expression profile of metabolism-related genes has great clinical value in various cancers^6^. For example, many metabolism-related genes can predict overall survival, indicate dysregulation of the metabolic microenvironment, and serve as potential biomarkers for metabolic therapy in gastric adenocarcinoma patients^7^. In addition, mutation-activated KRAS significantly increases glutathione synthesis and intracellular cysteine levels in lung adenocarcinoma. The construction of predictive models with metabolism-related genes based on the TCGA database can reveal the characteristics of the immune microenvironment of lung adenocarcinoma and predict patient prognosis^8,9^. Therefore, understanding the cellular and molecular mechanisms underlying tumour cell metabolic patterns can provide a helpful basis for understanding tumour pathogenesis and developing novel treatment.

MB is a metabolism-driven brain tumour^5,10^. Clinical 18FDG-PET studies have shown that MB patients with a poor prognosis exhibit active glycolytic metabolism and high glucose uptake rates^11^. In the SHH subtype of MB, the regulation of specific metabolic genes, including hexokinase 2 (Hk2) and fatty acid synthase (FASN), by Hedgehog (Shh) was found to shift the metabolic pattern toward aerobic glycolytic metabolism and lipid synthesis to maintain tumour growth^12^. Reducing lipid synthesis with FASN inhibitors slows tumour progression and prolongs survival^13^. Moreover, the pro-oncogene MYC drives cancer cell growth by altering cell metabolism, leading to glutathione accumulation in Group 3 MB, which is characterized by MYC amplification. The inhibition of glutathione production exerts a synergistic effect with carboplatin treatment^14^. Therefore, precise in-depth staging of MB from a metabolic perspective is worthwhile, and targeting specific metabolic enzymes and metabolic pathways may be an effective cancer treatment strategy.

Our study aimed to identify key metabolic regulators in paediatric MB by conducting a comprehensive bioinformatics analysis of MB sample data from the Gene Expression Omnibus (GEO) database, followed by subsequent validation in independent datasets. In addition, to determine the possible function of the polyamine metabolic enzyme ODC1 in MB, cellular aggregation, movement, Cell Counting Kit-8, invasion and immune cell chemotaxis assays were performed with MB cell lines in vitro. Targeting ODC1-mediated polyamine metabolism enhances immune cell infiltration in the tumour microenvironment, inhibits tumour cell dependence on polyamines, slows tumour growth and improves patient prognosis.

## Materials and methods

### Datasets

Three datasets, namely, GSE74195, GSE164677, and GSE85217, were obtained from the Gene Expression Omnibus (https://www.ncbi.nlm.nih.gov/gds). For the GSE85217 dataset, samples of patients with full survival data and who were under the age of 18 were retained for further examination. In addition, the external independent MB database from the CBTTC was downloaded from UCSC Xena (http://xena.ucsc.edu/). The metabolic-related genes (MRGs) were obtained from the KEGG database^15^.

### Identification of differentially expressed MRGs (DE-MRGs) and functional enrichment analysis

First, the R package “limma” was used for differential gene expression analysis. The thresholds for identifying differentially expressed genes (DEGs) were a log2-fold change > 0.5 and an adjusted p value < 0.05. The intersection between the DEGs and MRGs was considered to indicate DE-MRGs. Ultimately, 71 DE-MRGs were identified. The DE-MRGs were subjected to Gene Ontology enrichment and enrichment assessments via the “clusterProfiler” package. The protein‒protein interaction (PPI) network of the DE-MRGs was obtained from the STRING database and constructed with Cytoscape software.

### Cluster analysis

First, univariate Cox analysis was conducted to identify prognostic DE-MRGs, and 21 of the 71 DE-MRGs were identified as prognostic DE-MRGs that were significantly associated with overall survival (OS) in the GSE85217 paediatric MB dataset. The nonnegative matrix factorization (NMF) algorithm was subsequently used for cluster analysis to identify metabolic-related molecular subtypes based on the 21 prognostic DE-MRGs in the GSE85217 cohort.

### Establishment and verification of the predictive risk score model

As previously mentioned in sections “Identification of differentially expressed MRGs (DE-MRGs) and functional enrichment analysis” and “Cluster analysis”, 21 prognostic DE-MRGs were identified in the GSE85217 paediatric MB cohort. Then, the Lasso Cox regression technique was employed to reduce the number of dimensions in R software. Finally, lambda.min was used to diminish the dimension, and we obtained 12 prognostic DE-MRGs for the development of a predictive risk model. The equation for determining the risk score was as follows:

$$Risk\, score={\sum }_{1}^{12}\beta i*Expi$$

where β_i_ is the regression coefficient of gene i obtained from Lasso, and Exp_i_ is the expression level of gene i.

The CBTTC database contains MB samples and provides the relevant clinical and survival information. MB patients aged < 18 years were selected and included in the CBTTC paediatric MB cohort, which was used to validate the risk model based on the abovementioned risk score formula.

### Survival analysis

Kaplan–Meier (K-M) survival and univariate and multivariate Cox analyses were performed to estimate the prognostic value of the genes and risk scores via R packages (survival and survminer).

### Gene set enrichment analysis (GSEA)

Based on the median risk score, the paediatric patients with MB were divided into two groups: the high-risk group and low-risk group. Subsequently, differential gene expression analysis was performed by using the “limma” package in R software. The results were subsequently used to conduct GSEA. The gene sets of KEGG pathway and HALLMARKER were downloaded from the MisgDB (https://www.gsea-msigdb.org/gsea/index.jsp). GSEA was performed to assess the pathway and biological process enrichment of the high- and low-risk groups by using the “clusterProfiler” package. The terms with an absolute normalized enrichment score (NES) > 1 and an adjusted p value < 0.05 were considered significant.

### Immune infiltration analysis

The ssGSEA and ESTIMATE algorithms were used to evaluate immune cell infiltration in each paediatric MB sample by using the R packages “GSVA” (version 1.42.0) and “estimate” (version 1.0.13), respectively. The 28 immune gene sets were described in a previous study^16^. Based on the 28 gene sets, the ssGSEA algorithm was used to quantify the relative infiltration of 28 immune cell types in the tumour microenvironment of MB. The ESTIMATE algorithm returned four scores, namely, the ImmuneScore, StromalScore, ESTIMATEScore, and tumour purity score.

### Tumour stemness index (TSI) analysis

Malta and his colleagues have reported a machine learning method, called one-class logistic regression (OCLR), that is used to quantify the stemness of samples based on their transcriptome and other profiles^17^. Based on the OCLR algorithm, the TISs of the MB samples were calculated and used for further analysis.

### Chemotherapy response analysis

The responses of the MB samples to drugs were predicted by the “pRRophetic” package in R. Based on the Genomics of Drug Sensitivity in Cancer (GDSC) database, the IC50 value of drugs in the MB samples were determined and used for correlation analysis.

### Cell culture

The human Daoy (RRID: CVCL_1167) and D283 Med (RRID: CVCL_1155) MB cell lines were purchased from Abiowell Biotechnology; these cell lines were confirmed by STR analysis and were negative for mycoplasma infection. MB cell lines and normal human glial cells (HEB cell line) were incubated in MEM (Gibco, USA) supplemented with 10% foetal bovine serum (BI, Israel) and antibiotics (penicillin/streptomycin) in a humidified incubator in 5% CO_2_ at 37 °C.

### Transfection

Small interfering RNAs (siRNAs) targeting ODC1 were purchased from GenePharma (Shanghai, China) and verified by DNA sequencing. The siRNAs were transfected into Daoy cells using Lipofectamine RNAiMAX (Invitrogen, New York, USA) according to the manufacturer’s instructions. The ODC1 siRNA sequences used were as follows: sense, GGUUGGUUUCAGCAUGUAUTT; antisense, AUACAUGCUGAAACCAACCTT.

### RNA extraction, RT-PCR, and qRT-PCR

Total RNA was isolated from Daoy cells using TRIzol reagent (Thermo Fisher, USA). According to the instructions, 500 ng of RNA was reverse transcribed into cDNA using the HiScript III 1st Strand cDNA Synthesis Kit (Vazyme, Nanjing, China). Then, the cDNA samples were subjected to real-time quantitative PCR using qPCR Master Mix (Promega, USA) and analysed with a PCR instrument (ABI7500, USA).

The sequences of the ODC1-specific primers that were used were as follows: forward primer, TTTACTGCCAAGGACATTCTGG; reverse primer, GGAGAGCTTTTAACCACCTCAG.

### Migration and invasion analyses

Daoy cells were pretreated with siRNA and polymines (5 μm)/DFMO (5 mM) for 48 h. A migration assay (Corning, USA) was performed with a Transwell plate (24-well) following the manufacturer's instructions. In brief, Daoy cells (5 × 10^4^) in 100 μl of MEM were seeded in the upper chambers of the Transwell plate, and 800 μl of complete medium was added to the lower chambers. After incubating at 37 °C for 24 h, the chambers were removed, and the cells on the surface of the upper chamber membrane were removed with a cotton swab. The cells on the lower layer of the chamber membrane were fixed in methanol at room temperature for 15 min. Next, the chambers were removed, and the methanol was drained. Then, the cells were stained with 0.5% crystal violet (Beyotime, China) for 15 min, washed with PBS, and allowed to dry. For the invasion assay (Corning, USA), the cell density was adjusted to 5 × 10^5^, and another procedure similar to that described above was conducted according to the manufacturer’s protocols. Finally, all the migrated cells were imaged and analysed by a Cytation 5 (BioTek, USA).

### Cell colony formation experiments

Forty-eight hours after treatment with siRNA and polymines (5 μm)/DFMO (5 mM), Daoy cells (1500 cells/well) were seeded in a 6-well plate and cultured for 10 days. The medium was discarded, and the cells were carefully washed three times with PBS solution. The cells were stained with 0.5% crystal violet for 15 min after fixation with methanol for 15 min. Finally, the dye solution was slowly removed by washing with running water, and the cells were air-dried. The number of colonies containing more than 50 cells was counted by a Cytation 5 (BioTek, USA).

### Western blotting

For Western blot analysis, cells were harvested, washed three times with phosphate-buffered saline (PBS), lysed with immunoprecipitation (IP) lysis buffer (Thermo Fisher, USA) for 30 min on ice, and centrifuged for 15 min at 12,000×*g* at 4 °C. The protein concentrations of the supernatants were determined using a BCA protein assay kit. Whole-cell lysates were subjected to SDS-PAGE and transferred to nylon membranes. The membranes were blocked with 5% BSA for 1 h at room temperature and then incubated with primary antibodies against ODC1 (1:1000 dilution) and GAPDH (1:2000 dilution) at 4 °C overnight. The membranes were then washed with PBS-T three times and subsequently hybridized with peroxidase-conjugated secondary antibodies for 1 h at room temperature, followed by washing with PBS-T. Finally, the blots were developed in an Amersham Imager 600 (General Electric, USA).

### CCK-8 assay

Daoy cells were pretreated with siRNA and polymines (5 μM)/DFMO (5 mM) for 48 h and seeded in 96-well plates at a concentration of 4 × 10^3^ cells per well. After incubation at 37 °C for 24 h, cell counting kit-8 (APExBIO, USA) reagent was added to MEM at a 1:9 ratio. Then, 100 μl of this detection solution was added to each well, and the cells were incubated at 37 °C for 1 h. The absorbance of the solution was measured at 450 nm using Labsystems (Thermo Fisher, USA).

### PBMC chemotaxis assay

D283 Med cells (1.0 × 10^5^) were treated with or without polymines (5 μM)/DFMO (5 mM); then, the cells were seeded in the lower chamber of a Transwell plate (6-well, 8 μm) in 1.6 ml of MEM. PBMCs (1 × 10^6^) were stained with Cell Tracker Red at 37 °C for 45 min and seeded in the upper chamber of the Transwell plate in 400 μl of MEM. After the Daoy cells were cocultured with PBMCs for 72 h, the upper chamber was removed, and images of the lower chamber were captured and analysed by a Cytation 5 (BioTek, USA).

## Results

### Identification of differentially expressed metabolism-related genes and functional enrichment analysis

**Figure 1:**
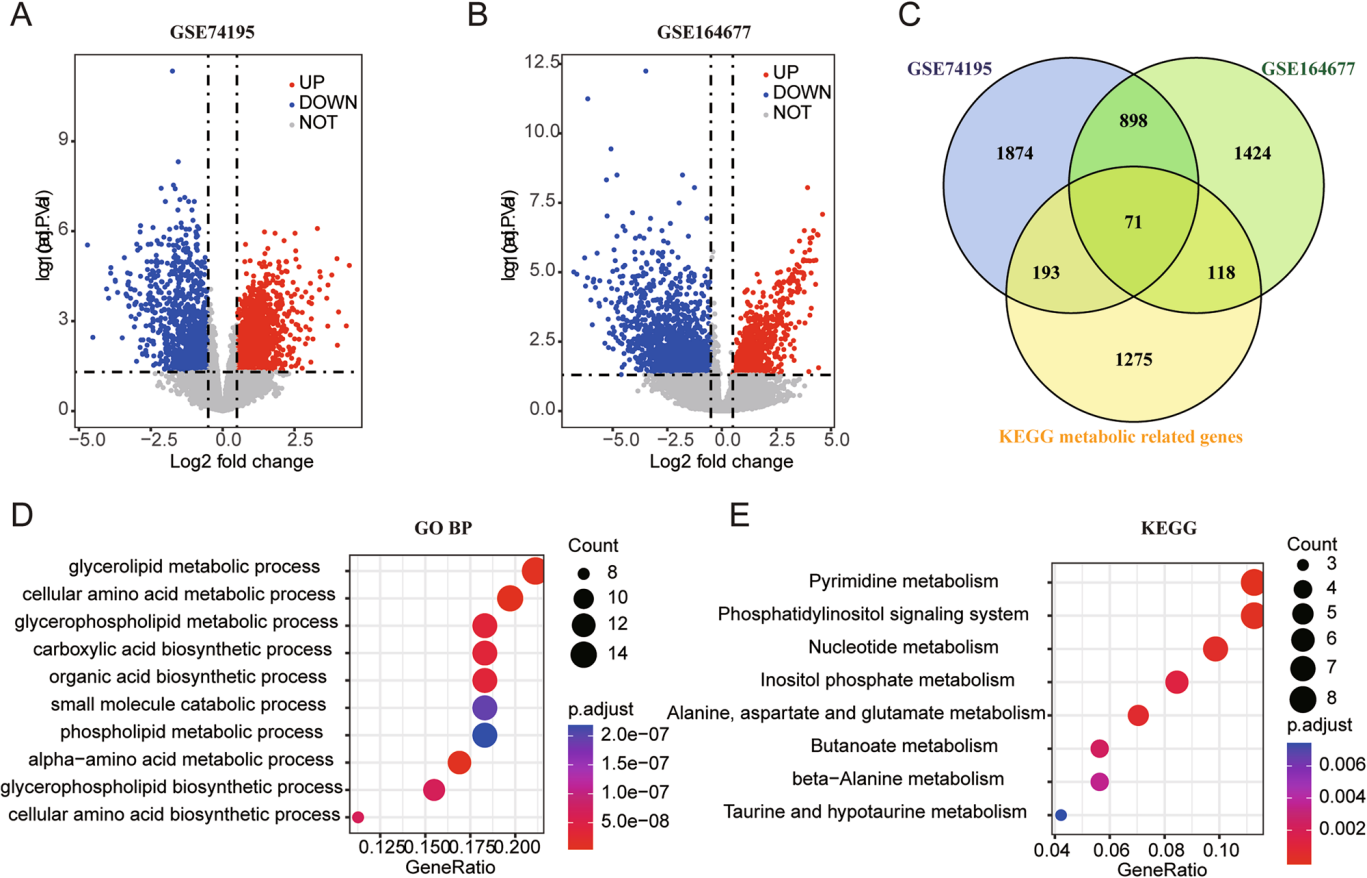
Identification of DE-MRGs in MB and functional enrichment analysis. (**A**) Volcano plot showed the DEGs between MB and cerebellum in the GSE74195 database. (**B**) Volcano plot showed the DEGs between MB and para-tumor in the GSE164677 database. (**C**) Venn graphic showed the 71 DE-MRGs. (**D**) Dot plot showed the top 10 enriched GO bp terms. (**E**) Dot plot showed the enriched KEGG pathways.

First, we identified 1657 metabolism-related genes based on the KEGG database. Differential expression analysis was subsequently performed to identify DEGs between MB tissue and normal tissue samples from the GSE74195 and GSE164677 datasets. Based on the preset thresholds for DEGs, there were 3036 DEGs in the GSE74195 dataset and 2511 DEGs in the GSE164677 dataset (Fig. 1A,B). After identifying common genes between the DEGs and MRGs, we identified 71 DE-MRGs in MB (Fig. 1C). To better understand the underlying mechanism of these 71 genes, GO and KEGG enrichment analyses were performed. The results showed that these genes were enriched mainly in various metabolic processes, lyase activity, and metabolic-related pathways (Fig. 1D,E). Additionally, the PPI network of these genes is shown in Fig. S1 and was constructed based on the STRING database.

### Identification of prognostic MRGs and metabolism-associated clusters

**Figure 2:**
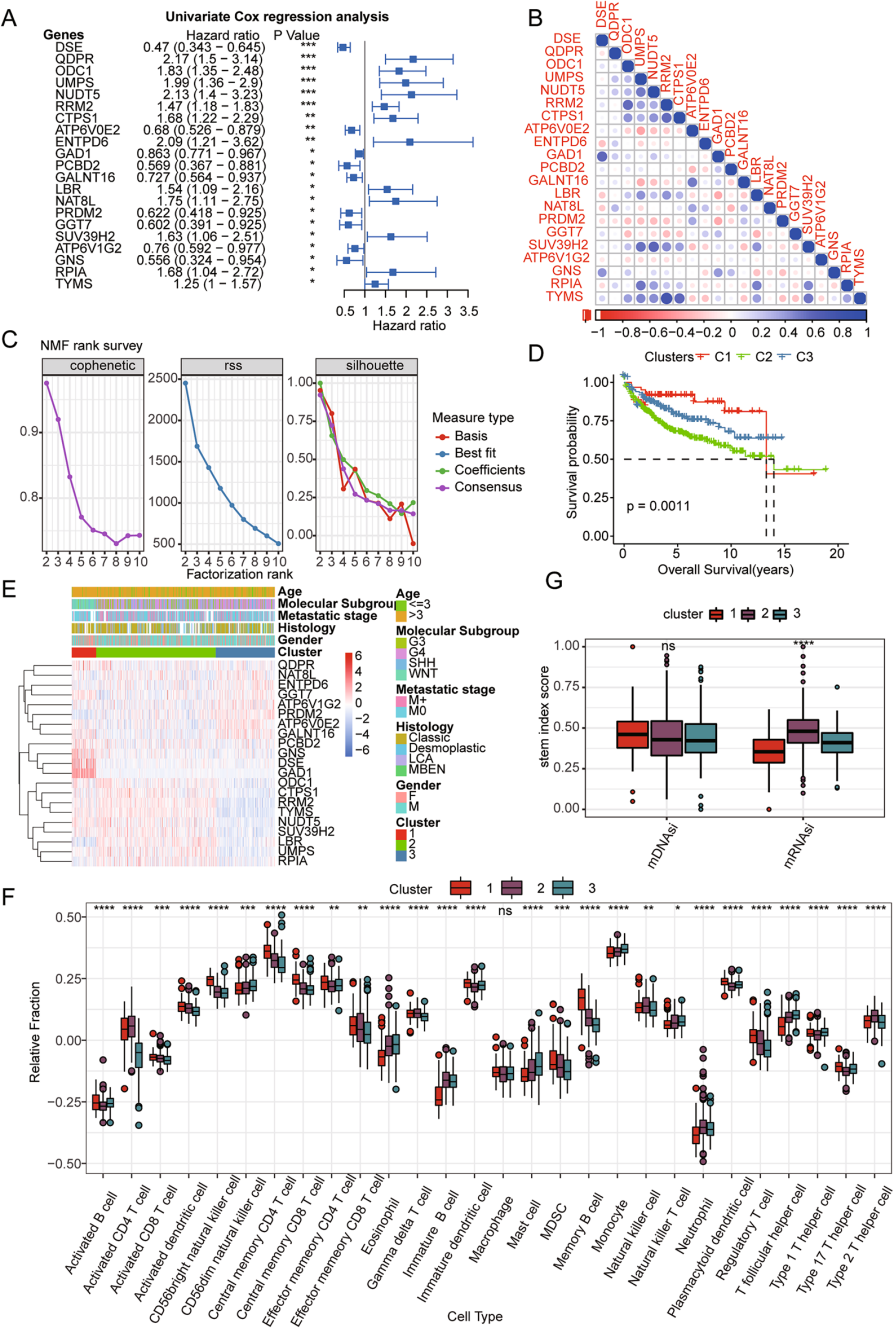
Identification of prognostic MRGs and metabolism-associated clusters. (**A**) Twenty-one of the 71 MRGs were significantly associated with the prognosis of paediatric patients with MB according to univariate Cox analysis. (**B**) Correlations among the 21 prognostic MRGs. (**C**) The relationships among cophenetic, dispersion, and silhouette coefficients with respect to the number of clusters. (**D**) K‒M survival curves of OS time of patients in the three clusters. (**E**) The heatmap of the expression patterns of the 21 MRGs in the clusters. (**F**) The difference in the degree of immune cell infiltration among the three clusters based on the ssGSEA algorithm. (**G**) The difference in stemness scores among the three clusters.

To identify prognostic DE-MRGs in paediatric MB, univariate Cox regression analysis was performed, and the results revealed that 21 of the 71 DE-MRGs (2 genes were unmatched) were significantly correlated with the OS of paediatric patients with MB in the GSE85217 cohort (Fig. 2A). The correlations among these 21 genes were analysed, and the results revealed that there were significant correlations among the expression levels of the majority of the genes (Fig. 2B). These 21 prognostic MRGs were further used to conduct cluster analysis of the GSE85217 paediatric MB cohort. Using the NMF algorithm, we identified three distinct paediatric MB clusters, namely, Cluster 1, Cluster 2, and Cluster 3 (Fig. 2C). To compare the prognoses among these clusters, K‒M survival analysis was performed, and K‒M curves were drawn. The results showed that the overall survival of these three clusters was significantly different: Cluster 1 had a good prognosis, followed by Cluster 2, and Cluster 3 had the worst prognosis (Fig. 2D). A heatmap was constructed to visualize the expression patterns of these 21 genes among these three clusters, and the results revealed that these genes were significantly associated with the age, metastatic stage, histology, and molecular subtypes of the MB patients (Fig. 2E). Furthermore, the ssGSEA algorithm was used to estimate the degree of infiltration of 28 immune cells in paediatric MB samples. Differential analysis revealed that immune cell infiltration was significantly different among these three clusters (Fig. 2F). In addition, we explored the differences in stemness indices among these three clusters. As shown in Fig. 2G, the difference in mRNAsi among the three clusters was significant, and the mRNAsi of Cluster 2 was the highest; however, there was no significant difference in mDNAsi among the three clusters.

### Construction and validation of a prognostic risk score model for paediatric MB

**Figure 3:**
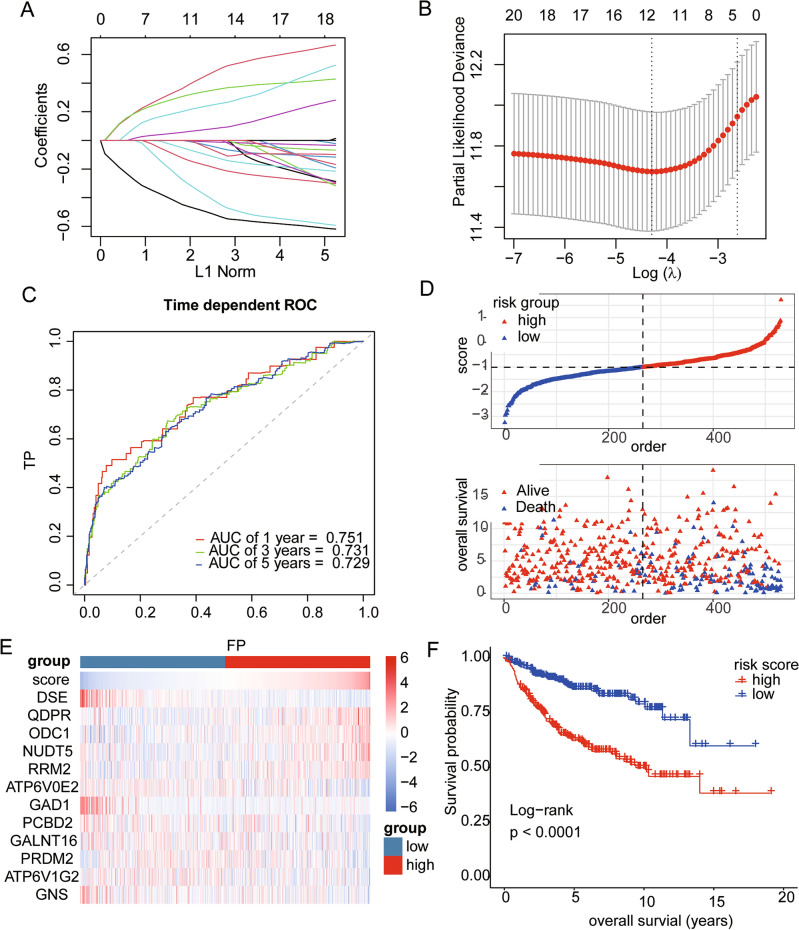
Construction of a prognostic risk model for paediatric MB. (**A**) The coefficient profiles of 21 MRGs based on the LASSO algorithm. (**B**) The selection of the optimal parameter (lambda) based on Ten-time cross-validation. (**C**) The time-dependent ROC curves of OS time based on the GSE85217 paediatric MB cohort. (**D**) The distributions of risk score, OS, and survival status in the GSE85217 paediatric MB cohort. (**E**) The heatmap shows the relationship between the risk score and the expression of 12 MRGs in this risk model. (**F**) K‒M curves of OS in high-risk and low-risk patients.

To construct a prognostic risk score model for paediatric MB based on the DE-MRGs, LASSO Cox analysis was conducted. Ultimately, 12 DE-MRGs, DSE, QDPR, ODC1, NUDT5, RRM2, ATP6V0E2, GAD1, PCBD2, GALNT16, PRDM2, ATP6V1G2, and GNS, were selected, and the coefficients of these genes were extracted (Fig. 3A,B). Based on the formula mentioned in the “Materials and methods” section, we computed the risk score for each MB sample. To assess the accuracy of this prognostic model in predicting OS, time-dependent ROC analysis was conducted, and curves of 1-, 3-, and 5-year OS were generated. The findings indicated that the area under the ROC curve (AUC) of this model for 1-, 3- and 5-year OS was 0.751, 0.731, and 0.729, respectively, which indicated that the signature of the 12 MRGs exhibited outstanding prognostic efficacy (Fig. 3C). Then, according to the median value of the risk score, paediatric MB patients were divided into a high-risk group and a low-risk group. Figure 3D shows the allocation of risk scores and survival status between the high-risk group and low-risk group based on the GSE85217 paediatric MB cohort. The gene expression levels of the 12 DE-MRGs in each paediatric MB patient were visualized in a heatmap (Fig. 3E). K‒M survival curves were generated and showed that paediatric patients with MB in the high-risk group had significantly shorter OS than did those in the low-risk group (Fig. 3F). Consistent with these findings, we obtained comparable results in the CBTTC-MB cohort, demonstrating that our risk model has exceptional efficacy in predicting the prognosis of paediatric MB patients (Fig. S2).

### The prognostic model is an independent predictor of prognosis in paediatric patients with MB

**Figure 4:**
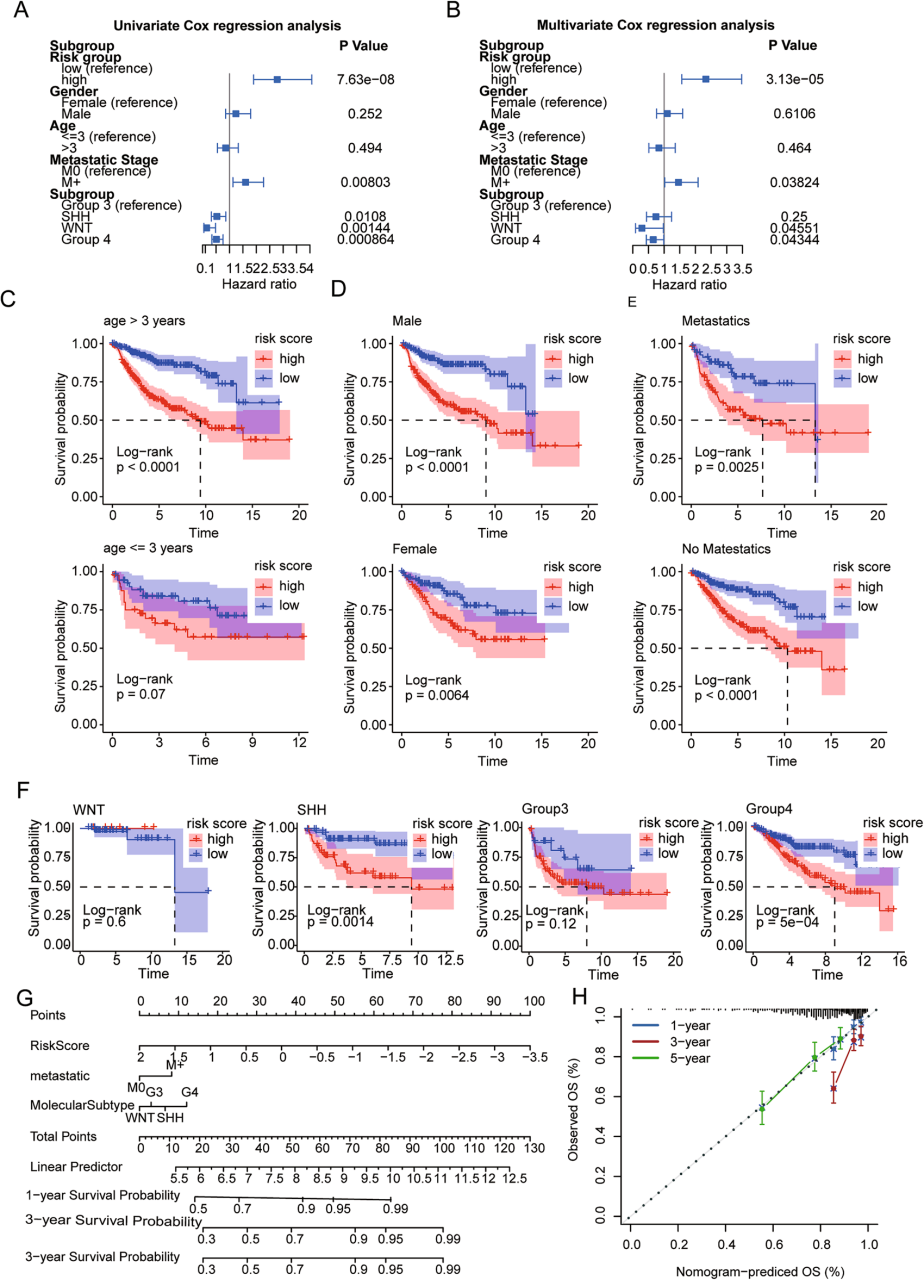
Cox survival and stratification analyses of the risk model in paediatric patients with MB. (**A**) The forest plot of univariate Cox analysis results based on the GSE85217 dataset. (**B**) The forest plot of multivariate Cox analysis results based on the GSE85217 dataset. (**C**) K‒M survival curves of patients in different age groups. (**D**) K‒M survival curves of patients of different sexes. (**E**) K‒M survival curves of patients with/without metastasis. (**F**) K‒M survival curves of patients in different molecular subgroups. (**G**) Nomogram based on the risk score, metastatic stage, and molecular subtype in the GSE85217 dataset. (**H**) Calibration plots of the nomogram for predicting the probability of 1-, 3-, and 5-year OS.

To determine whether the constructed prognostic model was an autonomous prognostic factor for paediatric MB, both univariate and multivariate Cox analyses were performed. Univariate Cox regression analysis of the GSE85217 dataset demonstrated that the danger score, metastatic stage, and molecular subtype were significantly associated with the prognosis of paediatric MB patients, whereas sex and age were not associated with the prognosis of paediatric MB patients (Fig. 4A). Multivariate Cox regression analysis of the GSE85217 dataset indicated that the risk score was an independent prognostic factor for paediatric MB after adjusting for the abovementioned clinical and molecular characteristics (Fig. 4B). Furthermore, stratified analysis grouped by sex, age, metastatic stage, and molecular subgroup was performed to confirm the robustness of our risk model. The findings revealed that individuals with lower risk scores consistently experienced longer overall survival than did those with higher risk scores in subgroups with different ages, sexes, metastatic stages, and molecular subtypes (Fig. 4C–F). In addition, risk score, metastatic stage, and molecular subtype were selected to construct a nomogram model based on the results of multivariate Cox analysis; among these factors, the risk score was the most important factor (Fig. 4G). According to the calibration plots, the OS times predicted by this nomogram model were highly consistent with the actual 1-year, 3-year, and 5-year OS times based on the GSE85217 paediatric MB cohort (Fig. 4H).

### Clinical and molecular characteristics of risk score

**Figure 5:**
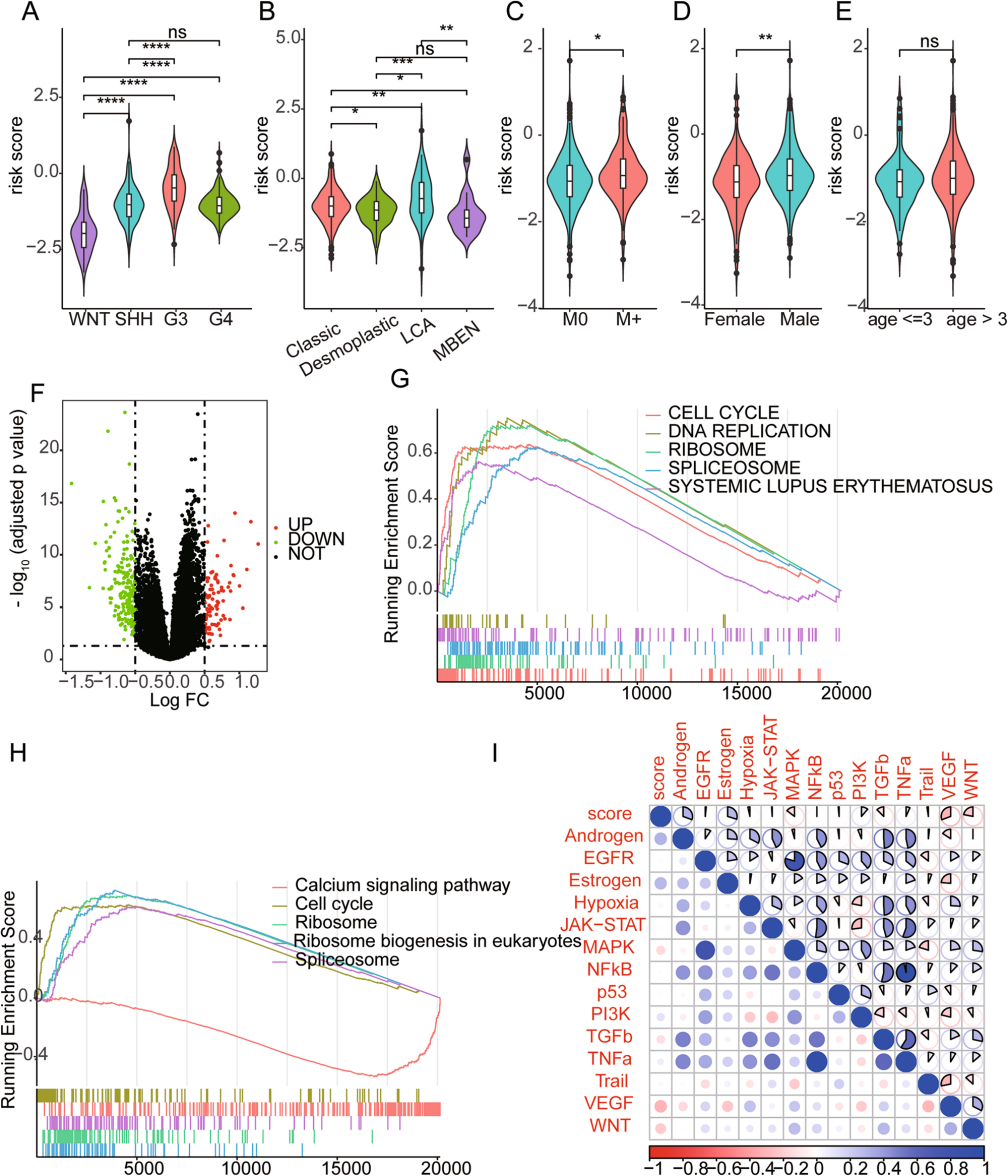
Clinical and Molecular characteristics of risk score. (**A**) Analysis of differences in risk scores among molecular subtypes. (**B**) Difference analysis of risk score among the histological subtypes. (**C**) Difference analysis of risk score between patients with metastasis or not. (**D**) Difference analysis of risk score between gender subgroups. (**E**) Difference analysis of risk score between age subgroups. (**F**) The volcano plot of DEGs between high-risk group and low-risk group. (**G**) The top 5 terms of enriched KEGG pathways. (**H**) The top 5 terms of enriched HALLMARKER pathways. (**I**) The correlation between risk score and 14 pathways activity.

We further examined the risk score of paediatric MB between different clinical and molecular subcategories. Our findings revealed that the risk score was significantly associated with molecular subtype (Fig. 5A), histology (Fig. 5B), metastatic stage (Fig. 5C), and sex (Fig. 5D). Nonetheless, no differences in risk score were observed among the age groups (Fig. 5E). To obtain a deeper understanding of the mechanism underlying differences in risk score, differential expression analysis was performed; 285 DEGs, including 86 upregulated and 199 downregulated genes, were identified between the high-risk group and low-risk group (Fig. 5F). GSEA was subsequently performed, and the results revealed that DEGs from the high-risk group were enriched in pathways related to the promotion of cancer, such as epithelial–mesenchymal transition, hypoxia, and KRAS signalling. Additionally, these genes were also enriched in pathways related to the immune response, such as the inflammatory response, cytokine‒cytokine receptor interaction, chemokine signalling pathway and focal adhesion. The top 5 pathways and terms are presented in Fig. 5G,H, respectively. In addition, we further analysed the correlation between the activity of 14 pathways and the risk score in paediatric MB and found that the risk score was positively correlated with the androgen, oestrogen, PI3K, and TNF-α pathways but negatively associated with the TNF-β, MAPK, VEGF, and WNT pathways (Fig. 5I).

### Risk score was correlated with immune cell infiltration and the stem index in MB

**Figure 6:**
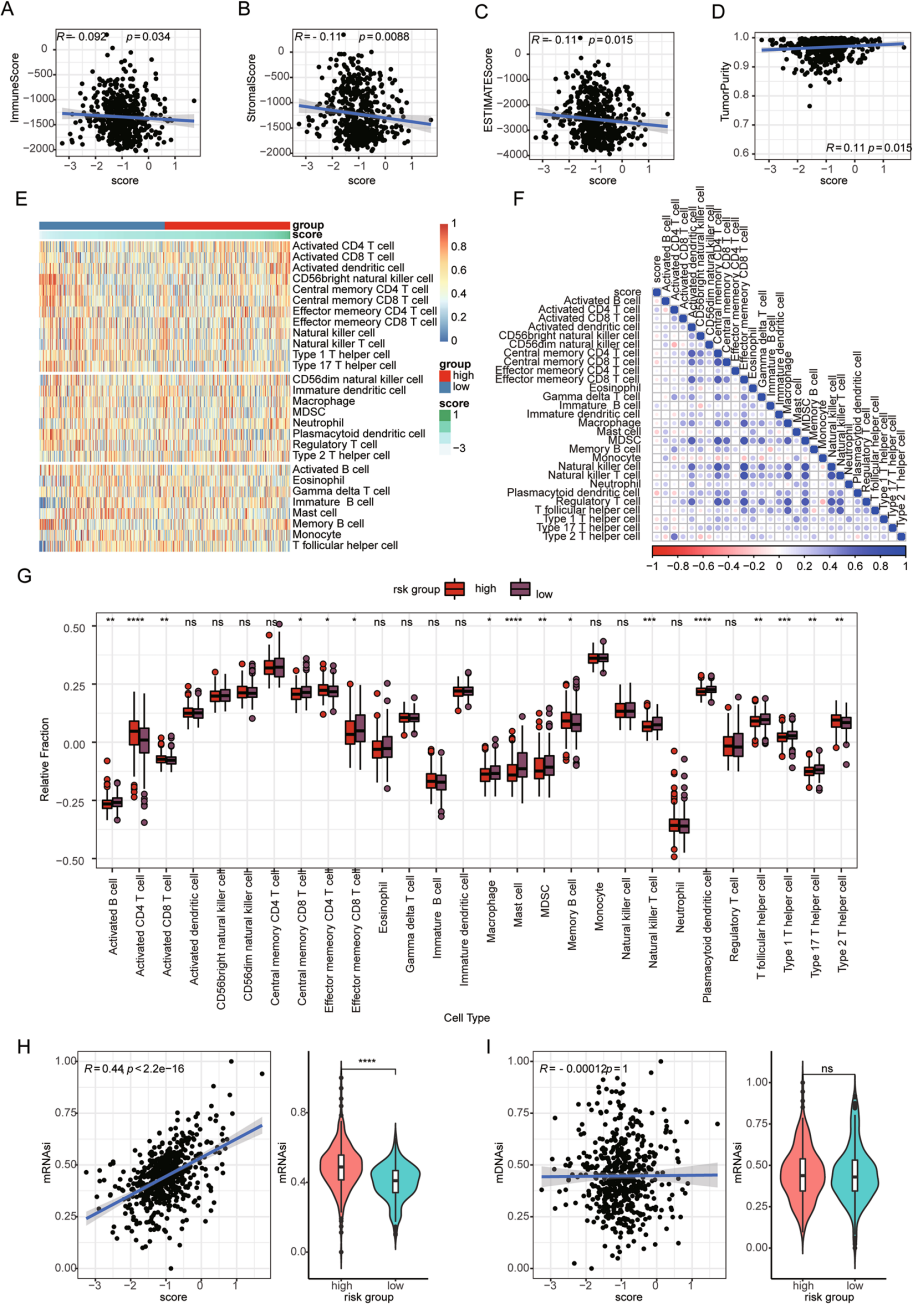
The risk score associated with the infiltration level of immune cells in MB. (**A**–**D**) The risk score significantly correlated with the immune score (**A**), stromal score (**B**), ESTIMATE score (**C**), and tumor purity (**D**). (**E**) The heatmap shows the infiltration level of 28 immune cells in low-risk group and high-risk group based on the ssGSEA algorithm. (**F**) The correlation between risk score and infiltration level of 28 immune cells. (**G**) The box plot shows the difference in infiltration level of 28 immune cells between high-risk group and low-risk group. (**H**,**I**) The association between risk score and stemness index, including mRNAsi (H) and mDNAsi (I).

As the functional enrichment analysis indicated an association between risk score and immune-related processes and pathways, we proceeded to investigate the correlation between risk score and tumour immune microenvironment in paediatric MB. First, the ESTIMATE algorithm was utilized to calculate the immune score, stromal score, ESTIMATE score, and tumour purity of each MB sample in the GSE85217 cohort. Correlation analysis was also conducted, and the results indicated that the risk score was negatively correlated with the three ESTIMATED scores but positively correlated with tumour purity (Fig. 6A–D). Moreover, we calculated the degrees of infiltration of 28 immune cell types in the GSE85217 cohort based on the ssGSEA algorithm. Our analysis showed that risk score was negatively correlated with the majority of immune cells in paediatric patients with MB (Fig. 6E,F). Differential analysis revealed significant differences in the degrees of infiltration of 16 immune cell types between the high-risk and low-risk groups (Fig. 6G). For most of these 16 cell types, the infiltration levels in the low-risk group were higher than those in the high-risk group according to the GSE85217 cohort (Fig. 6G). In addition, we calculated the correlation between risk score and mRNAsi and mDNAsi and assessed the difference in mRNAsi and mDNAsi between the high-risk group and low-risk group. The results showed that risk score was significantly positively correlated with mRNAsi and that mRNAsi in the high-risk group was significantly higher than that in the low-risk group (Fig. 6H); however, the correlation between risk score and mDNAsi was not statistically significant, and the difference in mDNAsi between the two risk groups was not significant (Fig. 6I).

### ODC1 regulates polyamine metabolism to promote tumor proliferation and invasion by modulating the immune microenvironment

**Figure 7:**
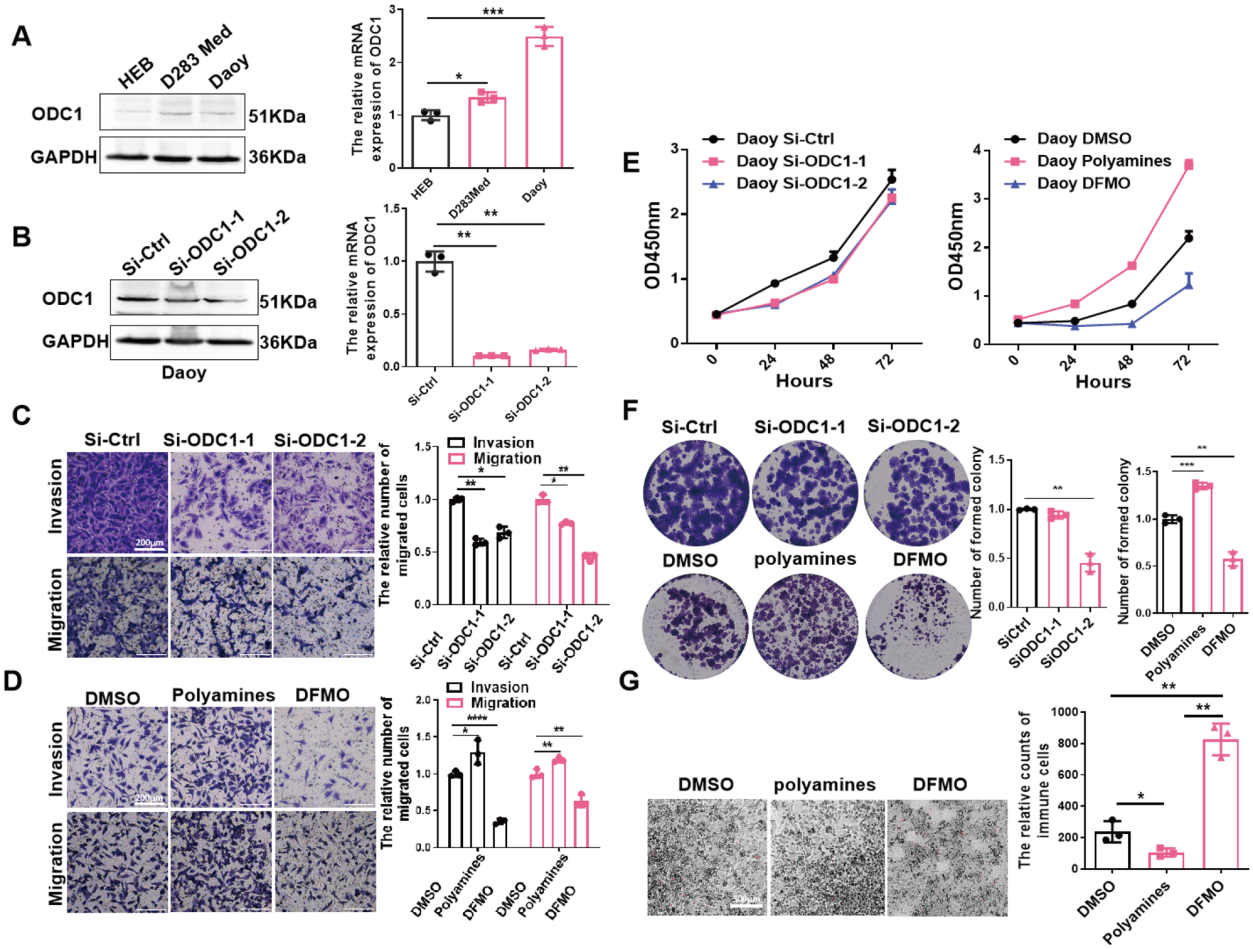
Validation of the role of polyamines and ODC1 on the proliferation, invasion, and migration of medulloblastoma. (**A**) Expression of ODC1 in medulloblastoma cell lines (Daoy and D283 Med) against human normal brain glial cells (HEB) was detected by WB and qPCR. (**B**) The specific siRNA downregulated the protein and mRNA level of ODC1. (**C**,**D**) The effect of ODC1 on the migration and invasion ability of Daoy cells was investigated by transwell assay. Representative images are shown on the left; scale bar: 200 μm. Quantitative analysis of cell migration and invasion levels was shown in the right panel. (**E**) Effects of ODC1 knockdown and addition of polyamines or DFMO on cell viability were determined by CCK-8 assay. (**F**) Proliferation ability of Daoy cells after ODC1 knockdown and polyamine supplementation was examined by colony formation assay. The left panel shows the overall colony formation in the entire culture dish. The right panel shows the number of cell colonies in each dish. (**G**) PBMC were co-cultured with D283 Med cells by transwell assay (0.4 μm) after labeling with cell tracker Red, and the number of PBMC cells chemotactic was observed under fluorescence microscopy.

Through previous analysis, we identified 12 MRGs that play important roles in paediatric MB. To validate the reliability of our bioinformatics analysis, we selected ODC1 and further studied its biological function in MB with experiments. Bioinformatics analysis revealed that ODC1 is highly expressed in MB and that its high expression predicts a poor prognosis. Compared to those in normal glial HEB cells, the protein and mRNA expression levels of ODC1 were notably upregulated in Daoy and D283 Med cells (Fig. 7A). ODC1 is a key rate-limiting enzyme in polyamine anabolism. It has been suggested that the occurrence of ODC1-mediated polyamine metabolism in MB and the accumulation of polyamines may drive tumour development. MBs are highly malignant brain tumours that readily metastasize to the dura mater via cerebrospinal fluid implantation. To further determine the role of ODC1-mediated polyamine metabolism in the proliferation and invasive metastasis of MB, we performed loss-of-function and gain-of-function analyses. First, we knocked down ODC1 in Daoy cells (Fig. 7B) and observed a significant decrease in the ability of tumour cells to invade and migrate (Fig. 7C). Since ODC1 is a key metabolic enzyme in the synthesis of polyamines, we added polyamines to Daoy cells; the results revealed enhanced cell migration and invasion, while DFMO drugs that were used to inhibit ODC1 had the opposite effect (Fig. 7D). In addition, we further verified by cell proliferation and colony formation assays that siRNA-mediated knockdown of ODC1 or DFMO drug-mediated inhibition of ODC1 expression decreased cell proliferation and colony formation, while the opposite was observed after in vitro polyamine supplementation (Fig. 7E,F). These results elucidate that enhanced expression of ODC1 in MB cells accounts for polyamine accumulation and promotes tumour cell proliferation and invasion. Chemotaxis experiments showed that polyamine compounds reduced the infiltration of PBMCs to Med283 cells (Fig. 7G). In addition, we compared differences in the degrees of infiltration of 28 immune cell types and the expression of immune checkpoint genes between groups with different ODC1 expression and polyamine metabolism activity. The results showed that the degrees of infiltration of most immune cell types, including NK cells, effector memory CD4 T cells and CD8 T cells, macrophages, mast cells, MDSCs, memory B cells and so on, in groups with high polyamine metabolism activity group and high ODC1 expression were significantly lower than those in the groups with low polyamine metabolism activity and low ODC1 expression (Fig. S3A,B). These results suggest that active polyamine metabolism in tumour cells may reduce NK and CD8 T cell infiltration and exert antitumour immunosuppressive effects.

Furthermore, we analyzed the relationship between the expression of ODC1 and the sensitivity to certain drugs. Figure S4 shows that the IC50 values of entinostat, FH535, GSK1904529A, KIN001-266, MetAP2.Inhibitor, CPI-163, Alisertib, Trichostati-A, Panobinostat, AGI-6780, Kobe2602, Vorinostat, Afatinib, Dihydrorotenone, I.BRD9, and telomerase inhibitor IX, were negatively correlated with high ODC1 expression, suggesting that patients with high ODC1 expression might benefit from these chemotherapeutic agents, which have low IC50 values, while patients with low ODC1 expression might benefit from CGP.60474 and BMS.754807.

## Discussion

MB is the most common malignant brain tumour in children; it mainly occurs in the posterior fossa and has the potential to spread through the cerebrospinal fluid. The histopathological classification is still the main system for categorizing MB and is extensively utilized in clinical practice^18^. However, prognosis varies greatly among patients with different MB subtypes. It is well known that metabolic reprogramming is an essential signature of tumour development. Many studies have demonstrated that abnormalities in metabolic enzymes, metabolic pathways, and metabolites facilitate the proliferation and invasion of tumour cells. Therefore, it is feasible to identify biomarkers of MB based on MRGs. In this study, we comprehensively analysed the prognostic value of MRGs, identified potential biomarkers and metabolic molecular subtypes, constructed a predictive risk model for MB, and further explored the biological function of ODC1 in MB based on in vitro experiments. Here, we categorized patients with MB into three groups according to the 21 prognostic MRGs by using the NMF algorithm. Patients in the three subgroups had different clinical prognoses, immune cell infiltration levels, and tumour stem indices. Furthermore, we developed and verified a predictive risk model utilizing 12 prognostic DE-MRGs that categorized MB patients into high- and low-risk cohorts. Patients in the risk subgroups also presented different clinical prognoses, immune cell infiltration levels, and tumour stem indices. Cox analyses, both univariate and multivariate, revealed that our risk model is an independent predictor of MB. Stratification analyses demonstrated that this risk model can identify patients with poor outcomes in different clinical, histopathological, and molecular subgroups. The MB patients in the high-risk group always had poorer prognoses than did those in the low-risk group. When we compared the C-index of the risk model to that of other parameters, we observed that the risk model had the highest C-index in both the GSE and ICTTB MB datasets. These results suggested that our risk model is effective in predicting the prognosis of MB patients with high accuracy and in identifying patients who need aggressive treatment.

Recently, increasing studies have revealed that metabolic reprogramming plays an important role in tumorigenesis and contributes to immune evasion^19,20^. The metabolic reprogramming of cancer cells can be indicative of resident and recruited cancer-associated stromal cells and immune cells in the tumour microenvironment (TME), which participate in regulating various processes and modulate the development of cancer^20^. In this study, we observed that our risk score was significantly associated with the degree of infiltration of immune cells in paediatric MB. The functional enrichment analysis indicated that our risk score was enriched for pathways related to the immune system. These results revealed that the 12 genes in our risk model may play important roles in regulating immune cells in the TME of MB through metabolic reprogramming. Furthermore, pathway activity analyses revealed that our risk score were correlated with pathways related to the promotion of, such as the androgen, oestrogen, PI3K, TNF-α, TNF-β, MAPK, VEGF, and WNT pathways. Several studies have indicated that oestrogen receptor β is expressed in MB and that its activation promotes MB cell proliferation and migration and inhibits apoptosis^21^. Belcher et al. reported that oestrogens reduce the sensitivity of MB to cisplatin^22^. Many studies have revealed that both the MAPK and PI3K signalling pathways are important pathways and act as key downstream effector factors or upstream modulators of many drives of MB metastasis^23^. VEGF signalling plays a critical role in tumour angiogenesis. Slongo et al. reported that VEGF and its receptors are expressed in human MB cell lines^24^. Some studies have reported that the VEGF signalling pathway participates in promoting MB cell growth, migration, and invasion^25^. In the present study, we discovered a significant correlation between our risk score and the metastatic status of MB. Therefore, these 12 genes might be involved in regulating MB metastasis.

The proposed signature included twelve metabolic genes (DSE, QDPR, ODC1, NUDT5, RRM2, ATP6V0E2, GAD1, PCBD2, GALNT16, PRDM2, ATP6V1G2, and GNS), and among these genes, high expression of four genes (QDPR, ODC1, NUDT5, and RRM2) was associated with poor prognosis, whereas elevated expression of the remaining eight genes (DSE, ATP6V0E2, GAD1, PCBD2, GALNT16, PRDM2, ATP6V1G2, and GNS) was associated with favourable outcomes in paediatric patients with MB. To the best of our knowledge, no previous investigations have described the use of these genes, other than RRM2, as predictors of OS in patients with MB. Many studies have indicated that most of these 12 MRGs are associated with various types of cancer. As the most important subunit of ribonucleotide reductase, RRM2 is the key regulatory enzyme for DNA synthesis and repair and is involved in many critical cellular processes^26^. Many studies have reported that RRM2 is overexpressed and acts as a tumour driver in various cancers, such as liver cancer^27^, lung cancer^28^, and breast cancer^29^. Guo et al.^30^ identified that RRM2 is a core gene in MB via integrated bioinformatics analysis. In this study, we observed that high RRM2 expression was markedly associated with unfavourable outcomes in patients with MB. DSE is a C5 epimerase and plays an important role in converting GlcA to iduronic acid (IdoA). Previous studies have demonstrated that DSE is overexpressed in several cancers, such as glioma, breast cancer, and hepatocellular carcinoma, and regulates growth factor signalling in cancer cells^31–33^. NUDT5, an ADP ribose pyrophosphatase, is associated with nucleotide metabolism and cancer. According to Zhang et al., breast cancer cells exhibit reduced growth and metastasis potential upon inhibition of NUDT5 through the AKT/cyclin D signalling pathway^34^. Glutamate decarboxylase 1 (GAD1) is the key enzyme that regulates the synthesis of GABA. Recently, many researchers have suggested that GAD1 is significantly overexpressed in various neoplastic tissues^35^. GAD1 knockdown inhibits the cellular invasiveness and migratory abilities of human oral cancer cells by regulating β-catenin translocation and MMP7 activation^36^. Zhang et al. demonstrated that miR-3174 promotes cell proliferation, accelerates the cell cycle, and inhibits cell apoptosis by targeting PCBD2 in rectal cancer^37^. The polypeptide N‐acetylgalactosaminyltransferase 16 (GALNT16) is involved in protein O‐glycosylation, and its polymorphisms are correlated with susceptibility to breast cancer^38^. PRDM2, a member of the histone/protein methyltransferase superfamily, is a tumour suppressor gene, and its expression is downregulated in some solid tumours, such as multiple myeloma and pituitary adenoma^39–41^.

Ornithine decarboxylase (ODC1) is a key rate-limiting enzyme that directly mediates the metabolism of polyamine production. Many studies have shown that an imbalance in polyamine synthesis is involved in the pathophysiological transformation of tumour cells, and large amounts of polyamines accumulate in rapidly growing tumour cells, such as breast cancer^42^, stomach cancer^43^, prostate cancer^44^, neuroblastoma^45^, and hepatocellular carcinoma^46^ cells. Polyamines, which include putrescine, spermine, and spermidine, are bioactive polycations that bind nucleic acids and proteins that regulate signalling pathways^47^. Recently, emerging studies have demonstrated that ODC1 is aberrantly expressed and plays a vital role in cancer. For example, Zi Ye^48^ reported that ODC1 was overexpressed in HCC and that silencing ODC1 attenuated cell proliferation, migration, and invasion. D’Amico et al.^49^ reported that ODC1 was elevated in MB and that difluoromethyl ornithine (DFMO) suppressed the proliferation of primary SHH MB cells. Consistent with these findings, we discovered that ODC1 was aberrantly overexpressed in primary (SHH subtype) and metastatic (Group 3 subtype) MB. Furthermore, we found that ODC1 was upregulated in both primary and metastatic MB cells. Silencing ODC1 through genetic and chemotherapeutic approaches inhibited the proliferation, migration, and invasion of MB cells. Moreover, the administration of polyamines decreased the immune infiltration of Med283 cells. Furthermore, bioinformatics analysis revealed that both ODC1 expression and polyamine metabolism activity were considerably correlated with the degree of immune cell invasion in MB. These results revealed that the metabolic enzyme ODC1 drives the proliferation and metastasis of MB cells by reshaping their metabolic phenotype. DFMO is a specific inhibitor of polyamine biosynthesis enzymes and inhibits tumour formation^50^. Clinical trials have shown that DFMO is highly effective in reducing polyamine levels at safe doses^51^. Therefore, ODC1 is a potential therapeutic target for MB.

## Conclusions

In this study, we constructed a 12-metabolic regulator (DE-MRG)-based model for predicting paediatric MB prognosis using comprehensive bioinformatics analysis. In addition, we revealed that ODC1-induced polyamine metabolism enhances the growth and invasion of MB cell lines in vitro. In summary, metabolism-related models could be promising prognostic methods for the clinical treatment of MB.

### Supplementary Information

Supplementary Information.

Supplementary Figures.

## Data Availability

The datasets used and analysed during the current study are available in the Gene Expression Omnibus (https://www.ncbi.nlm.nih.gov/gds) and UCSC Xena (http://xena.ucsc.edu/) repository.

## Abbreviations

MB: Medulloblastoma
DE-MRGs: Metabolism regulators
ODC1: Ornithine decarboxylase (ODC1)
Hk2: Hexokinase 2
FASN: Fatty acid synthase
MAPK: Mitogen-activated protein kinase
VEGF: Vascular endothelial growth factor
TME: Tumor microenvironment
RRM2: Ribonucleoside-diphosphate reductase subunit M2
DSE: Dermatan sulfate epimerase 1
NUDT5: NUDIX hydrolase 5
GAD1: Glutamate decarboxylase 1
PCBD2: Pterin-4 alpha-carbinolamine dehydratase 2
GALNT16: Polypeptide *N*‐acetylgalactosaminyltransferase 16
PRDM2: Positive regulatory domain zinc finger protein2
DFMO: Difluoromethyl ornithine
GSEA: Gene set enrichment analysis
HR: Hazard ratio
OS: Overall survival
GO: Gene ontology
LASSO: Least absolute shrinkage and selection operator
TCGA: The cancer genome atlas
GEO: Gene expression omnibus
