# Supplementary material for: Identification and validation of a metabolism-related gene signature for predicting the prognosis of paediatric medulloblastoma
Source: Sci Rep. 2024 Mar 30;14:7540. doi: 10.1038/s41598-024-57549-2 (PMC10980764; doi:10.1038/s41598-024-57549-2)
Supplement: Supplementary file 1 — Supplementary Information. [file 41598_2024_57549_MOESM1_ESM.docx]

Supplementary Materials for

**Identification and validation of a metabolism-related gene signature for predicting the prognosis of paediatric medulloblastoma**

Jun Su^1^, Qin Xie^2^, Longlong Xie^3^ *

1. Department of Neurosurgery, The Affiliated Children's Hospital Of Xiangya School of Medicine, Central South University (Hunan children’s hospital), No. 86 Ziyuan Road, Changsha 410007, Hunan, China
2. Department of Neurosurgery, Xiangya Hospital, central south university, No. 86 Xiangya Road, Changsha 410008, Hunan, China
3. Pediatrics Research Institute of Hunan Province, Hunan Provincial Key Laboratory of Pediatric Orthopedics, The Affiliated Children's Hospital Of Xiangya School of Medicine, Central South University (Hunan children’s hospital), No. 86 Ziyuan Road, Changsha 410007, Hunan, China.

Correspondence：xielonglong@hnetyy.net

**This PDF file includes:**

**Supplementary figure 1**

**
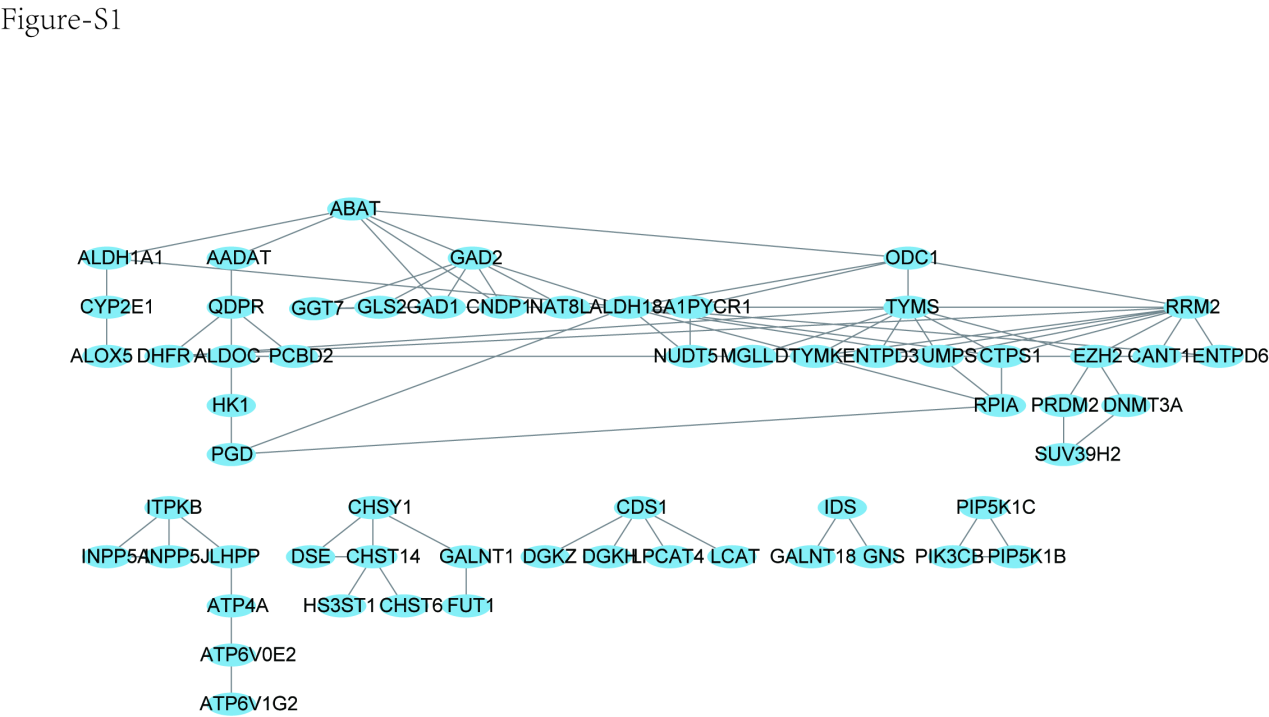
**

**Figure. S1: The PPI network of 71 DE-MRGs in MB**

**Supplementary figure 2**


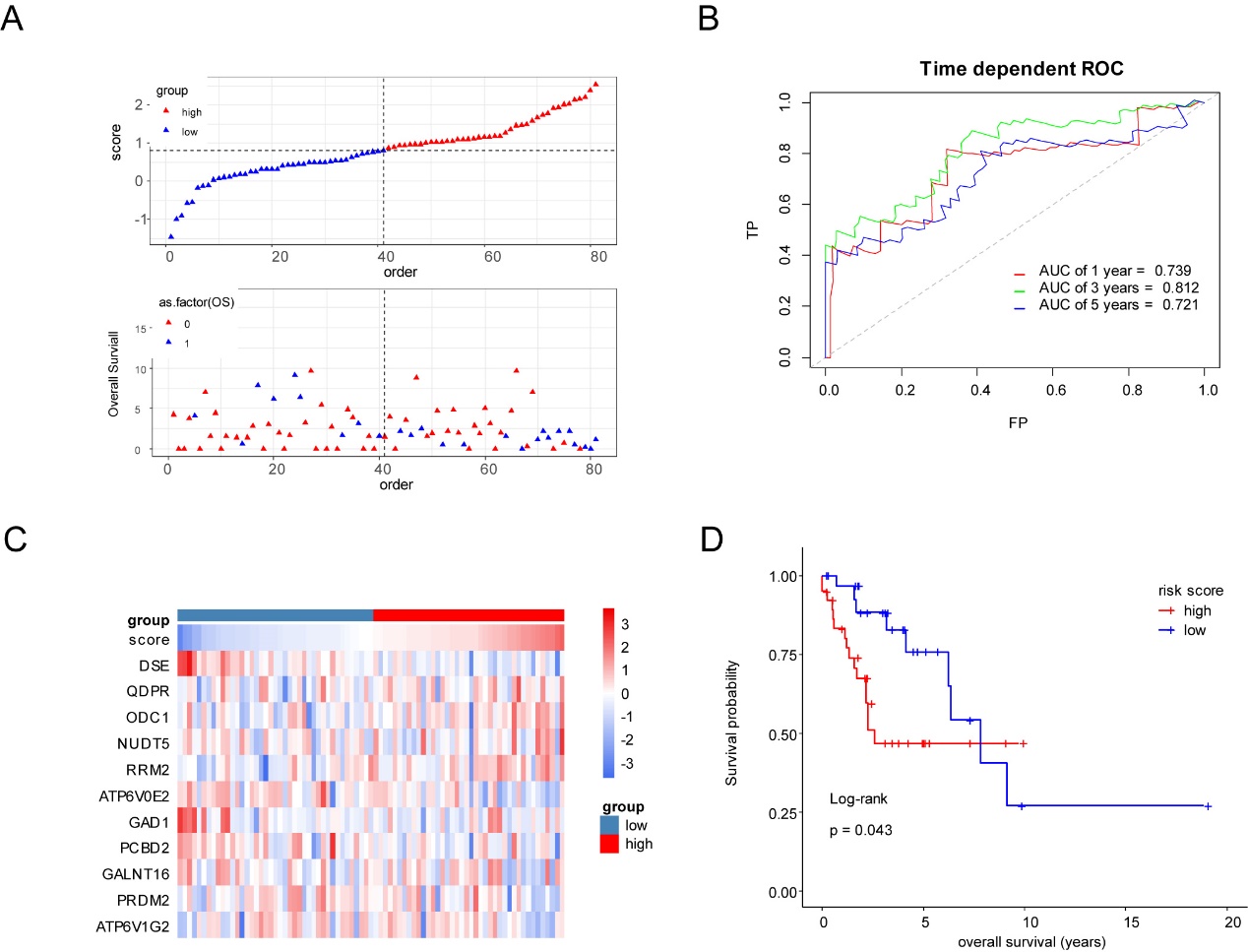


**Figure. S2 Validation of prognostic risk model for pediatric MB in testing cohort**

A The distribution of risk score, OS, and survival status in the CBTTC MB cohort.

B The time dependent ROC curves of OS time based on the in the CBTTC MB cohort.

C The heatmap shows the relationship between risk score and expression of 12 MRGs in this risk model.

D The K-M curves of OS time in high-risk and low-risk patients.

**Supplementary figure 3**

**
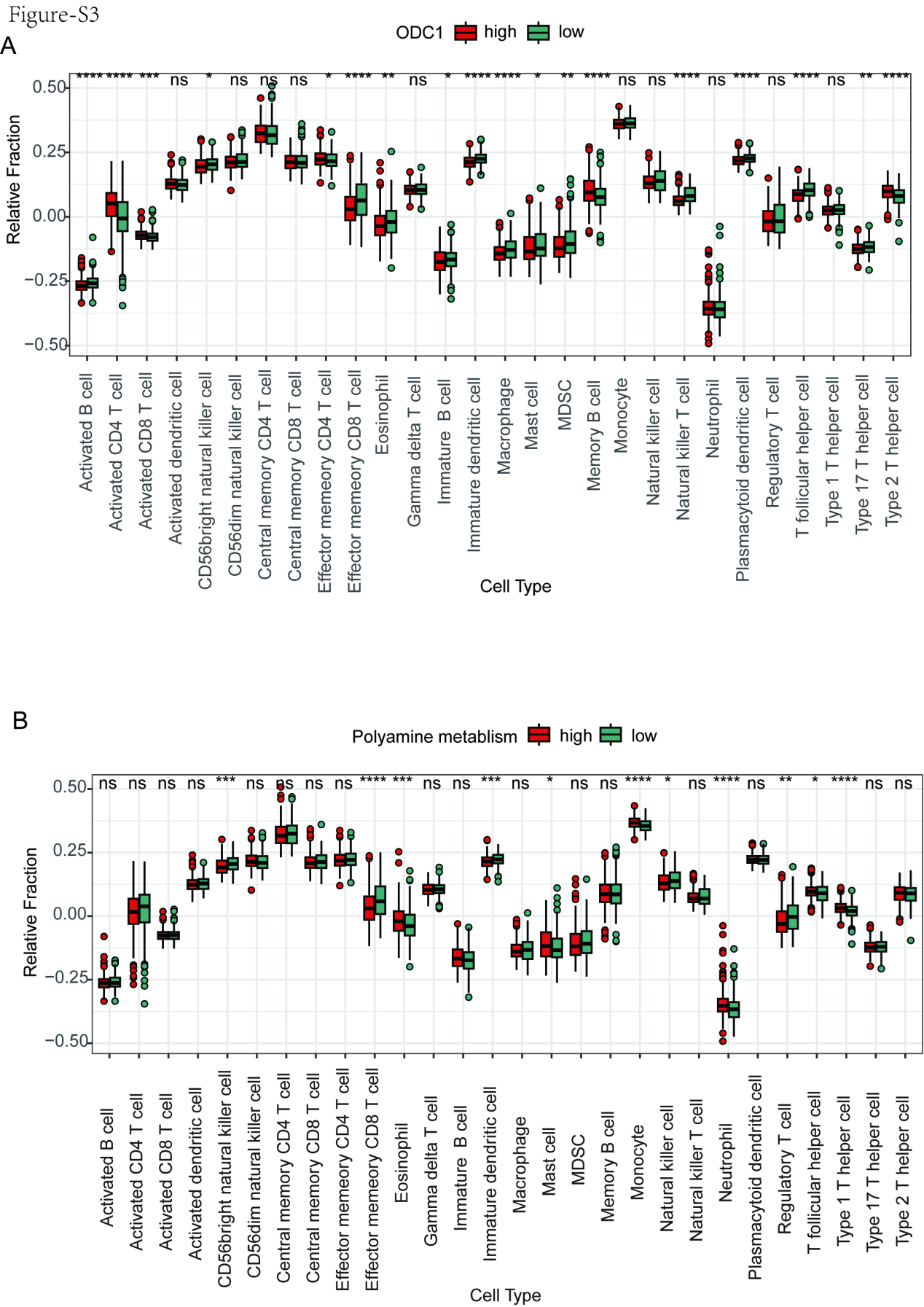
**

**Figure. S3 The ODC1 expression and polyamine metabolism activity associated with the infiltration level of immune cells in MB.**

A The box plot shows the difference in infiltration level of 28 immune cells between ODC high expression group and ODC1 low expression risk group.

B The box plot shows the difference in infiltration level of 28 immune cells between polyamine metabolism high activity group and polyamine metabolism low activity group.

**Supplementary figure 4**


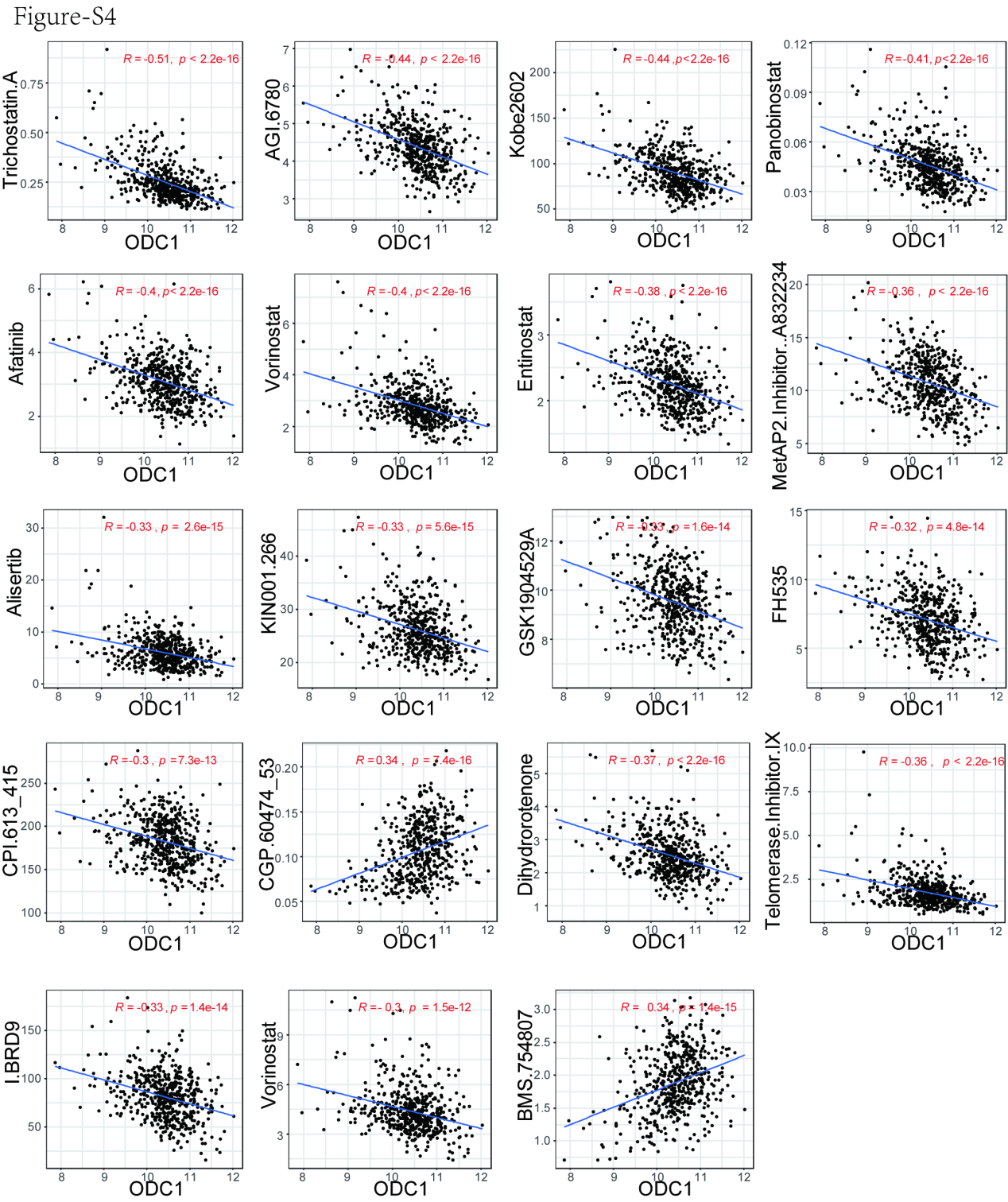


**Figure. S4 ODC1 expression corelated with sensitivity of some drugs.**
